# How Can Music Influence the Autonomic Nervous System Response in Patients with Severe Disorder of Consciousness?

**DOI:** 10.3389/fnins.2015.00461

**Published:** 2015-12-10

**Authors:** Francesco Riganello, Maria D. Cortese, Francesco Arcuri, Maria Quintieri, Giuliano Dolce

**Affiliations:** Research in Advanced Neurorehabilitation, Istituto S. AnnaCrotone, Italy

**Keywords:** disorder of consciousness, vegetative state, autonomic nervous system, central autonomic network, heart rate variability, music therapy, entropy

## Abstract

Activations to pleasant and unpleasant musical stimuli were observed within an extensive neuronal network and different brain structures, as well as in the processing of the syntactic and semantic aspects of the music. Previous studies evidenced a correlation between autonomic activity and emotion evoked by music listening in patients with Disorders of Consciousness (DoC). In this study, we analyzed retrospectively the autonomic response to musical stimuli by mean of normalized units of Low Frequency (nuLF) and Sample Entropy (SampEn) of Heart Rate Variability (HRV) parameters, and their possible correlation to the different complexity of four musical samples (i.e., Mussorgsky, Tchaikovsky, Grieg, and Boccherini) in Healthy subjects and Vegetative State/Unresponsive Wakefulness Syndrome (VS/UWS) patients. The complexity of musical sample was based on Formal Complexity and General Dynamics parameters defined by Imberty's semiology studies. The results showed a significant difference between the two groups for SampEn during the listening of Mussorgsky's music and for nuLF during the listening of Boccherini and Mussorgsky's music. Moreover, the VS/UWS group showed a reduction of nuLF as well as SampEn comparing music of increasing Formal Complexity and General Dynamics. These results put in evidence how the internal structure of the music can change the autonomic response in patients with DoC. Further investigations are required to better comprehend how musical stimulation can modify the autonomic response in DoC patients, in order to administer the stimuli in a more effective way.

## Introduction

Music listening is one of the most pleasurable experiences for the human being (Dube and Le Bel, 2003). Music can be defined as the organization of the tone over the time. By mean of the exposure to musical pieces in everyday life, listeners acquire sensitivity to the regularities of the tonal system (Tillmann, 2005). This knowledge creates expectancy in the listeners, with experience of tension, suspense or relaxation, when the rules are confirmed, or violated (Meyer, 2008; Ockelford, 2008). Activations to pleasant and unpleasant musical stimuli were observed within an extensive neuronal network of limbic and paralimbic brain structures. Activations in the ventral striatum, anterior superior insula, and in Rolandic operculum were observed in healthy subjects, during the listening of pleasant music (Koelsch et al., 2006). Moreover, inferior frontolateral cortex, ventrolateral premotor cortex, and anterior part of the superior temporal gyrus were found active in the processing of musical syntax, whereas the processing of musical semantics appears to activate posterior temporal regions (Koelsch, 2005).

Some studies also evidenced a correlation between autonomic activity (modulation of the High frequency component recorded by Heart Rate Variability), and emotion evoked by musical listening (Iwanaga et al., 2005; Orini et al., 2010).

The emotions felt by the listening to music were described as linked to the musical structures (Juslin and Sloboda, 2010). The parameters, defined as Formal Complexity and General Dynamics, provide informations about the relationship between musical structures and emotions (Imberty, 1976). Imberty defines Formal Complexity and General Dynamics, combining musical variables (as note duration, metric interval, density of notes per time unit, loudness, accents, syncopation and other characteristics of melodic, harmonic, and rhythmic structures) associating them to the emotion induced by the music (Imberty, 1976, 1997). In particular, the General Dynamics is defined as the number per time unit of notes played and their relative intensity, while the Formal Complexity as the intrinsic homogeneity of the musical structures (i.e., melodic recorsivity, rhythmic structure, dissonance etc; Table 1).

**Table 1:** **Descriptors of music formal complexity and general dynamics**.

**Formal complexity**	**General dynamics**
(Hm^*^t) ± (eI^*^eR)	V^*^I
(Hm^*^t) = structure homogeneity index (eI^*^eR) = Heterogeneity index Hm = melodic entropy computed on the epoch of music with melody t = duration of metric interval eI = mean variation in intensity of each note over the time eR = mean variation of duration	V = mean number of successive musical note per second I = subjective intensity

*Musical structure are characterized by means of the available descriptors of formal complexity and heterogeneity index (or intrinsic homogeneity; FC) and general dynamics (GD). FC describes the intrinsic organization and “predictability” of the music as to rhythm or melody or its lack of perceivable structure and is conventionally regarded as an index of emotional sympathy. GD describes the music in terms of volume, harmony, and rhythms and reportedly accounts for motor involvement. Different degree of FC and GD inducing to different emotional status*.

The emotional reactions to four musical samples of different complexity [Boccherini: Minuet; Grieg: Morning; Tchaikovsky: Pathetic (1st movement), and Mussorgsky: Night on bald mountain] were observed in Traumatic Brain Injured (TBI) patients and healthy subjects by mean of the Heart Rate Variability (HRV) analysis, with a classification of reported emotions by the normalized unit of the low frequency (nuLF; Riganello et al., 2008). Successively, it has been possible to infer positive and negative emotional responses in Vegetative State/Unresponsive Wakefulness Syndrome (VS/UWS) patients (Riganello et al., 2010a), undergone to the same experimental procedure, in particular when they were exposed to the Boccherini's music (positive emotion) and to the Mussorgsky's music (negative emotion).

In the HRV analysis, the data are analyzed in time, frequency, and non-linear domains (Task Force of the European Society of Cardiology the North American Society of Pacing Electrophysiology, 1996; Riganello et al., 2012b). In the time domain, HRV measures are mainly markers of overall HRV. Detailed informations on the HRV dynamics and frequency components are provided by the analysis in the frequency domain by Fast Fourier Transform (FFT) or autoregressive (AR) models (Malliani, 1999; Montano et al., 2009). The generalized frequency bands, in case of short-term HRV recordings, are the very low frequency (VLF: 0–0.04 Hz), low frequency (LF: 0.04–0.15 Hz), and high frequency (HF: 0.15–0.5 Hz). Specifically, normalized unit of Low Frequency (nuLF; Burr, 2007), computed as second step after the initial statistical estimation of the power in the Low Frequency and High Frequency bands [nuLF = LF/(LF+HF)], is deemed indicative of sympathovagal balance.

All frequency domains analyses are based on the recognition of certain predetermined patterns (in FFT the pattern is a sinusoidal wave). A possible alternative to characterize the variability of heart rate is to measure the regularity (or complexity) of the fluctuations. The entropy (non-linear analysis metrics) is a general approach to quantify the regularity or information content of the data, providing “hidden information” related to underlying mechanisms (Richman and Moorman, 2000). The Approximate Entropy (ApEn; Pincus and Goldberger, 1994) determines the conditional probability of similarity between a chosen data segment and the next set of segments of the same duration. ApEn has been developed for measuring the complexity of relatively short time series and the calculations are not based on specific assumptions regarding the internal structure or dynamics of the system. However, ApEn has some known shortcomings, such as bias, relative inconsistency, and dependence on the sample length. Sample Entropy (SampEn) reduces the bias of ApEn (Aboy et al., 2007) is more consistent and easier to compute than ApEn, and provides a more reliable estimate of the complexity of a signal. Moreover, it may be used for considerably shorter time series than the ApEn, (<200 points; Batchinsky et al., 2009).

Heart rate complexity data (entropy) reflect overall balance of autonomic outflow, responsiveness, and neuroendocrine mechanisms (Ryan et al., 2011; Riganello et al., 2012a,b). Decreased variability is thought to reflect system isolation and a reduced ability to respond to perturbations. Entropy analysis represents potential powerful methods to use in the care for critically ill patients. In Intensive Care Unit reduced entropy was associated with illness and predicts death (Papaioannou et al., 2006, 2008; Riordan et al., 2009). The changes of entropy rates have been mainly related to aging and disease (Kaplan et al., 1991; Voss et al., 1996). More, it has been suggested that the complexity of short-term recording of heart rate variability might be closely related to cardiac autonomic modulation (Porta et al., 2000).

The decreased entropy of heart rate complexity, associated with Lifesaving Interventions in both prehospital trauma (Cancio et al., 2008) and Cardiac Autonomic Neuropathy patients, suggests a reduced responsiveness of the cardiac control mechanism to external and internal stimuli (Khandoker et al., 2009). The reduced entropy was significantly associated with an increase of mortality, and a relationship between entropy and death was found in patients with isolated severe head injury and with penetrating mechanisms of injury (Riordan et al., 2009).

Reported studies put in evidence the effectiveness of HRV analysis related to the autonomic nervous system (ANS) modifications. The children, who progressed to brain death, had a markedly lower LF/HF ratio, while the patients with more favorable outcomes had significantly higher LF/HF ratios (Biswas et al., 2000). As reported, a worsening of the conditions in TBI patients was correlated to the LF, the severity of neurological dysfunctions and the outcome, as well as the global HRV and parasympathetic tone were found higher in TBI patients, successively died (Goldstein et al., 1996; Rapenne et al., 2001; Norris et al., 2006). On the contrary, an amelioration was correlated to the recovery of autonomic functions, with a decrease of the parasympathetic activity, and a parallel recovery of the consciousness (Keren et al., 2005; Wijnen et al., 2006).

Many studies evidenced the different responses of the ANS, recorded by the HRV analysis, due to the different music styles. Different complex heart dynamics responses were observed, during the listening of different Indian Raga musics, assuming possible different responses based on the different musical patterns (Mukherjee et al., 2015). The effects of Iranian music on the cardiac function has been also studied (Hajizadeh et al., 2015), showing increasing values in the SampEn. Other studies suggest that excitative music decreases the activation of the parasympathetic nervous system in healthy subjects (Iwanaga et al., 2005), as well as the excitatory heavy metal music acutely decreases global HRV (da Silva et al., 2014). Exploring different styles of “relaxing” music, the “new age” music induced a shift in HRV from higher to lower frequencies, independently on the music preference of the listener (Perez-Lloret et al., 2014).

Previous studies (based on the analysis of the first 300 heartbeats recorded) have been designed to verify the possibility to classify positive or negative emotions elicited by different musical stimuli selected for their General Dynamics and Formal Complexity (Riganello et al., 2008), and their possible emotional effect in VS/UWS patients (Riganello et al., 2010a). The aim of this study was to verify the influence of the musical stimuli complexity on the autonomic responses in VS/UWS patients, by the HRV nuLF and SampEn parameters analysis during the listening of the first 3 min of the selected musical samples. The two musical samples (Boccherini and Mussorgsky; Riganello et al., 2010a) have been compared by the nuLF and SampEn parameters, taking in account the possible different effect observed in the VS/UWS patients. The hypothesis is that music with high Formal Complexity and General Dynamics reduces the autonomic response in VS/UWS patients.

## Materials and methods

**Figure 1:**
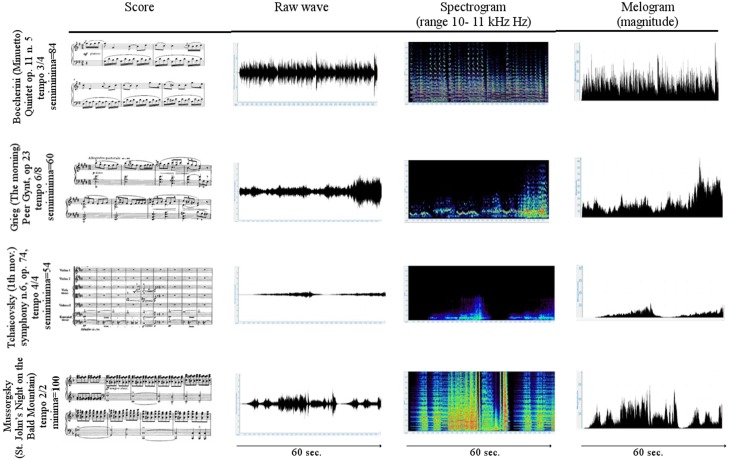
**Scores and first 60 s of the raw waveform, spectrogram, and melogram of the selected music**. In the 1st column the first bars of the musical score. In the 2nd column the raw wave formgraph displays the unprocessed recorded sound. In the 3rd column the spectrogram plot show the intensity of the frequency content of a signal as it changes over the time. The vertical axis indicates frequency, the horizontal axis indicates time, and the color or gray scales indicate the intensity. Magenta is the color of the highest and dark blue of the lowest intensity. In the 4th column the melogram (or magnitude) graph displays the intensity of the melody relative to a reference sound.

**Figure 2:**
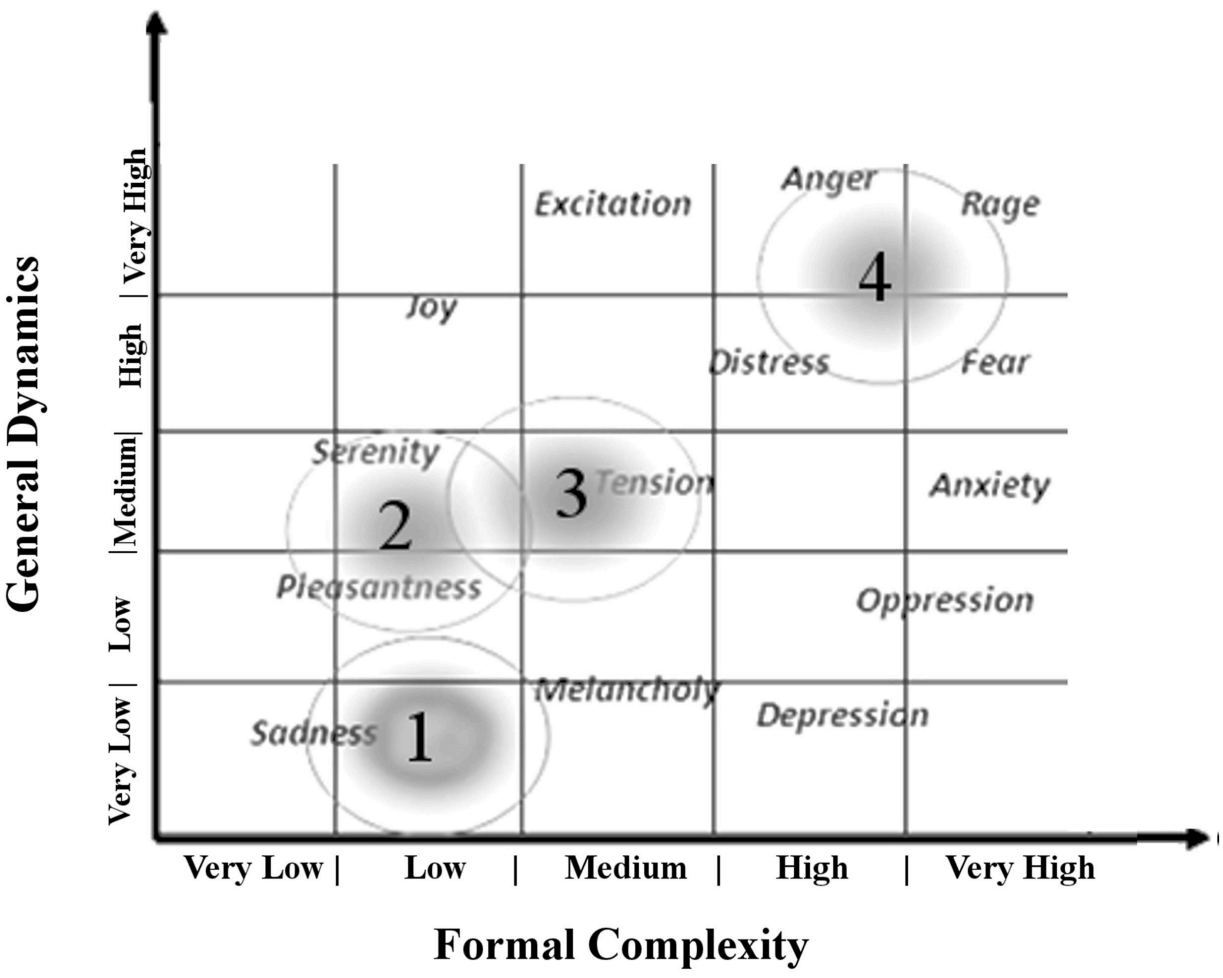
**Distribution of reported emotional responses of healthy subjects according to the music ***general dynamic*** and ***formal complexity*** (see Table 1)**. Numbers indicate the position in summary outline of the music samples used as auditory stimuli in the study (1 = Tchaikovsky; 2 = Boccherini; 3 = Grieg; 4 = Mussorgsky). Figure adapted from Riganello et al. (2010a).

The first 3 min of tachogram (i.e., the series of consecutive intervals between heartbeats) recorded during the listening of four musical samples [Boccherini (Minuet); Grieg (The morning); Tchaikovsky (Pathetic—1st movement); and Mussorgsky Night on bald mountain (Figure 1)], by Healthy subjects and VS/UWS patients, were retrospectively analyzed by Kubios HRV version 2.2 (Tarvainen et al., 2014). The selection was characterized by the Formal Complexity and the General Dynamics of the musical samples, as indicated by Imberty (Imberty, 1976, 1997; Juslin and Sloboda, 2010; Table 1; Figure 2). These descriptors are related to the musical structure and allow to characterize the (induced) emotional status along a continuum from euphoria and well-being to melancholy, severe anxiety etc. In particular an increasing of the Formal Complexity is associated to a major shift from “positive” toward “negative” emotions. In general, positive and negative emotions are associated to relatively simple or complex melodies and regular or irregular rhythms, respectively (Vitz, 1966; Crozier, 1974; Holbrook and Anand, 1990; Smith and Melara, 1990); slow and fast tempi are related to the ratings of subjective sadness and joy (Hodges, 1980; Gabrielsson and Juslin, 1996; Juslin, 1997, 2000). A matrix of the music Formal Complexity and General Dynamics was designed to classify the emotional responses and correlate them with the musical structures (see for detail Riganello et al., 2008; Figure 2).

Sixteen healthy subjects (9 women, 24–42 years, mean 31.8 ± 5.2) listened passively the music one time, while 9 patients (6 male, 16–31 years, mean:26 ± 6.0; 3 female, 31–48 years, mean: 39 ± 9) in VS/UWS (Table 2) according to current criteria (Giacino et al., 2004; Giacino and Kalmar, 2005), listened the music twice. All patients were assessed by Coma Recovery Scale Revised (CRS-R) administered by an expert neuropsychologist. The experiments took place always at the same time of the day (within 60 days from the injury), in the semi-intensive care unit dedicated to the vegetative state (the VS/UWS subjects' usual environment) and did not interfere with the patients' medical/rehabilitative schedule. VS/UWS subjects and healthy controls were comfortably lying on armchair, with constant 24°C ambient temperature and in absence of transient noises. The baseline was recorded at rest; subjects were exposed binaurally to the four selected music samples, presented via earplugs, balanced for loudness and played in random sequence to minimize carried-over effects. The music samples were presented in randomized sequence with 10 min of interval between each other. The VS/UWS patients were exposed to two music samples per day only in order to avoid overstimulation and excessive fatigue (for procedures detail see Riganello et al., 2010a).

**Table 2:** **Demographic data of VS/UWS patients**.

	**Age**	**Sex**	**Etiology**	**CRS-R**	**CRS-R sub scores**	**Follow-up at 6 month**
1	16	male	traumatic	6	A = 1 V = 2 M = 1 O = 1 C = 0 Ar = 1	MCS
2	21			5	A = 0 V = 1 M = 1 O = 1 C = 0 Ar = 2	VS
3	30			4	A = 0 V = 1 M = 1 O = 1 C = 0 Ar = 1	dead
4	30			5	A = 1 V = 1 M = 1 O = 1 C = 0 Ar = 1	VS
5	31			5	A = 1 V = 1 M = 1 O = 1 C = 0 Ar = 1	VS
6	27			6	A = 1 V = 1 M = 2 O = 1 C = 0 Ar = 1	VS
7	31	female	hemorrhagic	4	A = 0 V = 1 M = 1 O = 1 C = 0 Ar = 1	VS
8	39			6	A = 1 V = 2 M = 1 O = 1 C = 0 Ar = 2	VS
9	48			5	A = 0 V = 1 M = 2 O = 1 C = 0 Ar = 1	dead

*CRS-R subscore: A, Auditory Scale; V, Visual Scale; M, Motor Scale; O, Oromotor/Visual Scale; C, Communication Scale; Ar, Arousal Scale*.

The tachogram was analyzed in the time and frequency domains, by the HRV advanced analysis software developed at the Department of Applied Physics, University of Kuopio, Finland (Niskanen et al., 2004). The HRV nuLF and SampEn parameters were extracted for the analysis. Each couple of listening of the same musical sample administered to the VS/UWS patients, was averaged in one to avoid the error of alpha inflation in the sample size.

Healthy subjects vs. VS/UWS patients were compared between them for baseline and musical samples by Mann-Whitney's exact test. Difference among music samples was analyzed by Friedman's exact test and difference between Boccherini and Mussorgsky's music was analyzed by Wilcoxon's exact test in both groups. The exact test (Siegel, 1956; Gibbons and Chakraborti, 2011) is more accurate in case of small sample, or when the tables are sparse or unbalanced (Tanizaki, 1997; Mundry and Fischer, 1998; Gibbons and Chakraborti, 2011). The effect size (*r*) i.e., the index measuring the magnitude of difference or change between two conditions, (Rosenthal, 1991) was calculated as the *z*/square root (*N*; where *N* is the number of observations on which *z* is based) and will be hereafter formally referred to as not relevant (*r* < 0.1), small (0.1 < *r* < 0.3), medium (0.3 < *r* < 0.5), or large (*r* > 0.5; Hemphill, 2003). After the Bonferroni's corrections for multiple comparisons the results of the tests was considered significant for *p*-value ≤ 0.005.

The ethical principles of the Declaration of World Medical Association (2001) by the World Medical Association were followed.

## Results

After Bonferroni correction no significant difference was found in the baseline condition between groups.

The values of nuLF were found different between groups for Grieg (Mann-Whitney's exact test: *Z* = −2.887, *p* = 0.001, *r* = 0.58) and Mussorgsky's music (Mann-Whitney's exact test: *Z* = −3.170, *p* = 0.000, *r* = 0.63), while after Bonferroni correction, SampEn was found different only for Mussorgsky's music (Mann-Whitney's exact test: *Z* = −3.453 *p* = 0.000, *r* = 0.69).

**Figure 3:**
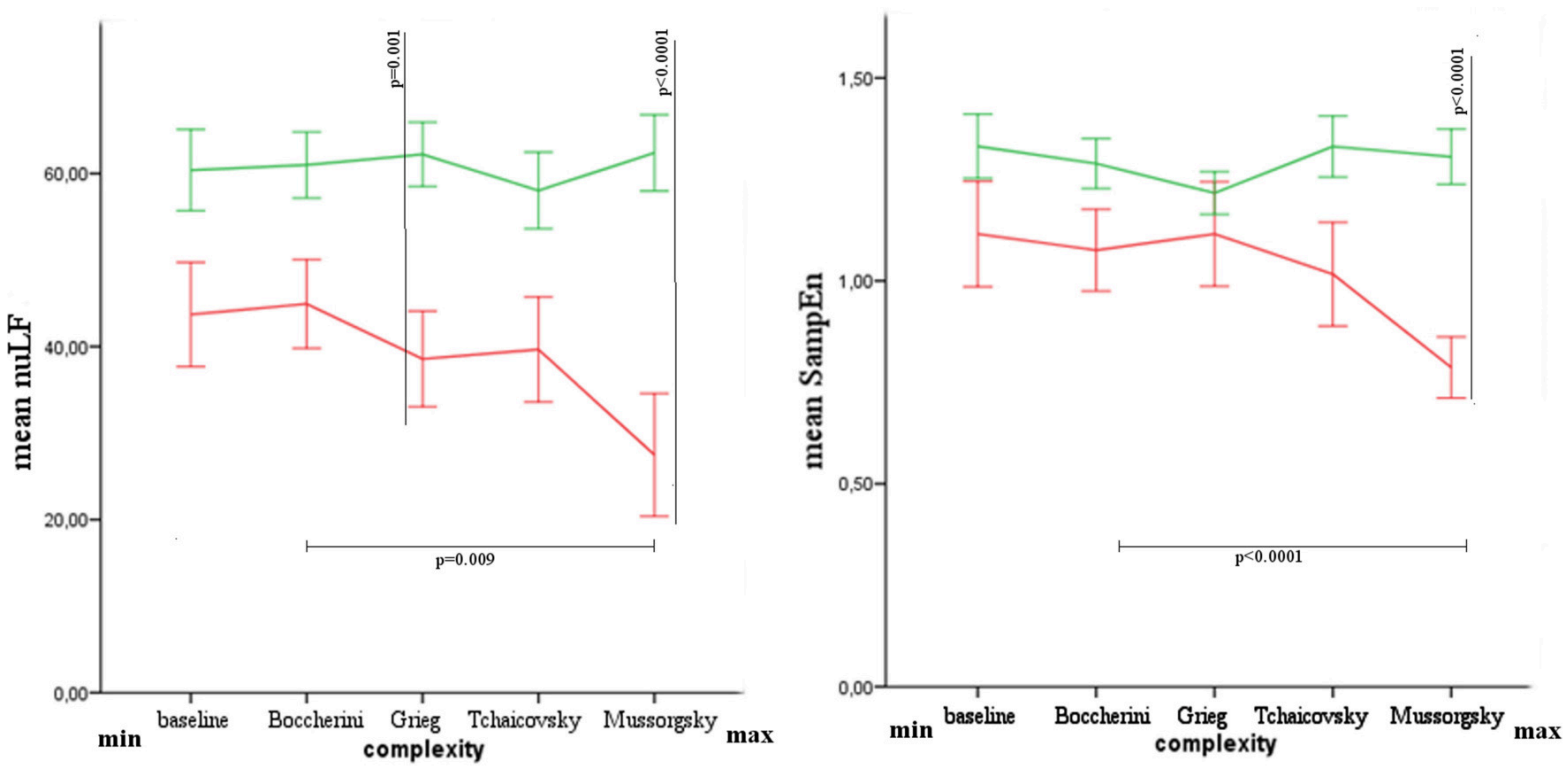
**Mean and Standard Error of nuLF and SampEn in healthy (green) and VS/UWS (red) groups**. In the axis, from left to right, baseline and musical stimuli with increasing complex structure.

The difference among musical stimuli was significant in VS/UWS group for nuLF (Friedman's exact test: χ^2^ = 10.733, *p* = 0.009) and SampEn (Friedman's exact test: χ^2^ = 16.067, *p* = 0.000) parameters. Comparing Boccherini and Mussorgsky's music, after Bonferroni correction, a significant difference was found for SampEn (Wilcoxon's exact test: 2.668, *p* = 0.002, *r* = 0.63; Figure 3).

## Discussion

The HRV analysis, of the previous study, was based on the first 300 heartbeats rate recorded (Riganello et al., 2010a), with different times of recording related to the subjects variability. The listening of Boccherini, compared with the other composers, showed a decreasing of the hearth rate. More, it was observed a difference in the nuLF between the baseline and the music samples listening. The nuLF was linked, by data mining analysis, to the emotions evoked by the different music (Riganello et al., 2008, 2010a), in particular too high or too low values of nuLF were associated to negative emotions.

In this study we tried to identify possible variations of the autonomic reactivity, specifically to the complexity of the musical stimulus, by analyzing the first 3 min of listening. The results were linked to the same length of the musical stimulation time, unlike the previous study, in which the HRV analysis was dependent also on the length of the tachogram.

The results showed differences between groups for HRV parameters, characterized generally by lower values in VS/UWS patients. This difference was significant for nuLF during the listening of Grieg and Mussorgsky, and for SampEn during the listening of Mussorgsky. Comparing the musical stimuli among them, the autonomic response was characterized by decreasing values in nuLF and SampEn in VS/UWS, when the musical complexity was higher. Such patients showed a shift of HRV parameters toward an increasing of vagal response, and contextually a reduction of heart rate complexity for increasing Formal Complexity and General Dynamics parameters. The different response to the musical stimuli was significant for SampEn, comparing Boccherini and Mussorgsky's musical samples. No similar response was observed in healthy subjects.

The experience of music listening is based on the idea that the music represents and induces emotions, which are, respectively, perceived and felt by listeners, although these two aspects may not coincide (Gabrielsson, 2002). The association of different psychological mechanisms, associated to the physiological correlates of the music listening, were suggested (Harrer and Harrer, 1968). Several modes of music listening were described as associated to conscious (e.g., structural analytic, associative oriented. ect.) or unconscious (e.g., associative emotional, motor-kinetic, etc.) listening (Rauhe, 1975; Rösing, 1985; Behne, 1986). As reported, the music internal structure plays a primary role in the induction of emotions, and rhythmic aspects are considered the major determinants of physiological responses (Gomez and Danuser, 2007). More, the tonal variation was correlated to the psychophysiological happy/sad distinction (Khalfa et al., 2008).

It was shown that the applications of music in medicine can be used to stabilize vital signs and manage symptoms in the short-term (Hanser, 2014). The listening of classical music and of rock music or noise were related to a small variance or an increase/decrease of Mayer Wave components and Respiratory Sinus Arrhythmia components, respectively (Umemura and Honda, 1998). Relaxation and music therapy have been found effective modalities to reduce stress and anxiety in patients of a coronary care unit (Zimmerman et al., 1988; Guzzetta, 1989; Hanser, 2014). Music therapy enhanced parasympathetic activities and decreased Congestive Heart Failure by reducing plasma cytokine and catecholamine levels (Okada et al., 2009).

Changes in the HRV patterns of response indicative of enhanced activity of the cardiovascular system were observed after 14-day music therapy (Lee et al., 2011). Replicable changes in the sympathovagal balance have been identified in DoC patients during the passive listening of symphonic music (Riganello et al., 2008, 2010a,b), allowing to cluster the autonomic responses as indicative of positive or negative emotions in both VS/UWS and awake posttraumatic subjects. Music appears peculiarly efficient in promoting arousal and responsiveness in DoC (O'Kelly et al., 2013). The activation of the superior temporal gyrus, by music, can predict the evolution from VS/UWS (Okumura et al., 2014).

The heart rate reflects the sympathetic/parasympathetic interplay, with a dominant tonus at rest of parasympathetic nervous vagus innervating the intrinsic cardiac nervous system (Scherlag and Po, 2006), and projecting to the sinoatrial node, atrioventricular node, and atrial cardiac muscle. ANS mediates the bidirectional communication between heart function and the Central Nervous System (CNS; Kawashima, 2005; Riganello et al., 2014). This regulation depends on medullar centers, in particular the nucleus of solitary tract and rostroventrolateral medulla (Spyer and Gourine, 2009) that integrate sensory information from proprio-, chemo-, and mechanoreceptors and from the telencephalic and limbic systems. An increase of rate results from reduced vagal activity (Hainsworth, 1995) or sympathetic activity above the intrinsic levels operated by the sinoatrial node (Hainsworth, 1995). An integrated model has been proposed (usually referred to as the Central Autonomic Network, CAN; Benarroch, 1993), in which neuronal structures involved in cognitive, affective, emotional, and autonomic regulation are functionally linked to heart function. This complex brain-heart interaction with the bidirectional links between cortical, midbrain, and brainstem structures (Riganello et al., 2012a) include, among others, the orbitofrontal, ventromedial prefrontal, anterior cingulate, and insular cortices, basal ganglia, central nucleus of the amygdala, nucleus of the solitary tract, nucleus ambiguus, and periaqueductal gray matter. The interplay between autonomic control and the CNS is modeled as a functional setup connecting through feedback and feed-forward loops the brainstem solitary tract nucleus with forebrain structures (Napadow et al., 2008; Lane et al., 2009; Riganello et al., 2012a).

Most of studies on HRV and music have been experimental rather than interventional, reporting significant changes in HRV as a function of musical mood (Etzel et al., 2006), genre (Bernardi et al., 2006), familiarity (Iwanaga et al., 2005), or tempo(Ellis, 2009; Fukumoto and Matsuo, 2010). Few reports exist of musical interventions that have included HRV as an index of autonomic function (Kemper et al., 2008; Okada et al., 2009; Ellis and Thayer, 2010; Roque et al., 2013a,b), and very few on musical stimuli and HRV in VS/UWS patients (Riganello et al., 2010a,b; Yen et al., 2010; Lee et al., 2011). However, it was suggested that the effect of music on cerebral processes in patients might reflect its capacity to act both on the external and internal neural networks supporting consciousness (Perrin et al., 2015). Again, it has been shown the benefit of classical and meditation music on patients hospitalized in intensive care medicine, whereas heavy metal or techno music were found not only ineffective, but possibly dangerous (Trappe, 2012). In these frames, it is important to define the correlations between musical structures and autonomic response to the musical stimuli, in order to have a correct approach to the stimulation of patients with DoC.

## Conclusions

The music listening is a complex experience and the responsiveness to the musical stimuli is constituted by a strong individual variability (Hanser, 2014). However, the study of the musical parameters can help to define and make hypothesis about musical stimuli and modification of ANS.

The close relationship between the CAN structures and the music listening could play an important function in the use of music in DoC patients (Magee, 2005; O'Kelly and Magee, 2013; O'Kelly et al., 2013). Complex musical stimuli could reduce the effectiveness of the response too. In order to improve the approach by the musical stimulation, further investigations are required to better comprehend how the musical structures can modify the autonomic response in DoC patients.

## Author contributions

All Authors equally contributed to the study design and preparation of the protocol as well as to the manuscript preparation. FR also performed the statistical analyses. The work reported in this paper has not been published previously, is not under consideration for publication elsewhere, and if accepted will not be published elsewhere including electronically in the same form, in English or in any other language, without the written consent of the copyright-holder. Its publication is approved by all authors and by the responsible authorities where the work was carried out. All Authors are employees of the Institute S. Anna–RAN and the study was supported by the Institute itself, without external funding.

### Conflict of interest statement

The authors declare that the research was conducted in the absence of any commercial or financial relationships that could be construed as a potential conflict of interest.
